# Pelvic plexus block to provide better anesthesia in transperineal template-guided prostate biopsy: a randomised controlled trial

**DOI:** 10.1186/s12894-019-0496-y

**Published:** 2019-07-09

**Authors:** Xue-fei Ding, Tian-bao Huang, Sheng-ming Lu, Hua-zhi Tao, Xiao-fang Ye, Fei Wang, Yao-zong Xu, Jia-nan Xu, Yu-quan Zhou, Yang Luan

**Affiliations:** 1 Department of Urology, Clinical Medical College, Yangzhou University, No. 98 West Nantong Road, Yangzhou, Jiangsu Province, 225001 China; 2 Department of Urology, Taihe County People’s Hospital, Fuyang, 226600 Anhui Province China

**Keywords:** Prostate, Biopsy, Nerve block, Pain

## Abstract

**Background:**

To evaluate the efficacy of pelvic plexus block (PPB) in relief pain during transperineal template-guided prostate biopsy (TTPB), compared with conventional periprostatic nerve block (PNB).

**Methods:**

From July 2016 to August 2017, 245 patients who were performed TTPB in Clinical Medical College of Yangzhou University were recruited. The patients were randomized into three groups using a random number table. Group-1 received prostate capsule local anesthesia with 22 ml of 1% lidocaine. Group-2 additionally received PNB on the basis of Group-1. To perform PNB, 5 ml 1% lidocaine was injected into the region of prostatic neurovascular bundle situated in the angle of prostate-bladder-seminal vesicle. Group-3 received prostate capsule local anesthesia plus PPB (5 ml of 1% lidocaine injection into the pelvic plexus which located on lateral to the bilateral seminal vesicle apex). The patients’ pain and satisfaction were evaluated by visual analogue scale and visual numerical scale, respectively.

**Results:**

The age, total prostate volume, PSA and the number of cores were comparable among the three groups. The visual analog scale scores of group-3 were significantly lower than group-2 during biopsy (*P* = 0.003). Conversely, the visual numeric scale scores were higher in group-3 (*P* = 0.039). Both the group-2 and group-3 outperformed the group-1 in alleviating pain and had a higher quantification of satisfaction. There were no significant differences in the pain scores or the satisfaction scores at 30 min after the procedure among the three groups.

**Conclusions:**

The analgesic efficacy of PPB guided by Doppler ultrasound in TTPB was better than that of PNB and both were superior to no nerve block.

**Trial registration:**

ChiCTR-IOR-17013533, 01/06/2016.

## Background

Early screening for prostate cancer (PCa) can significantly lower disease-related morbidity and even prolong life expectancy [1]. Transperineal template-guided prostate biopsy (TTPB) is one of most effective procedures for detection of prostate cancer [2, 3]. However, compared with the transrectal biopsy, transperineal route may cause more pain [4]. In the absence of anesthesia, about 20% of patients refused to undergo prostate biopsy [5]. Therefore, effective anesthesia is a important precondition for successful prostate biopsy [6]. Periprostatic nerve block (PNB) has attracted more and more attention due to its excellent analgesic effect and high safety. At present, PNB is regarded as the “golden standard” of analgesia during prostate biopsy [7–10]. In 1996, Nash et al. [11] were the first to introduce PNB, and found that it can relieve the pain during transrectal ultrasound (TRUS) -guided prostate biopsy. Conde et al. [12] found that the anesthesia effect of PNB was superior to oral morphine. Hergan et al. [13] conducted a meta analysis of fourteen trials and found that PNB had better anesthesia effect than that of local anesthesia or placebo. However, some patients have poor response to PNB in clinical practice. Apart from sensitivity to pain, most of these patients were with larger prostate volume [14]. It may be that, due to the increase in prostate volume may lead to a decrease in prostate cancer detection rate [15]. Hence, in order to improve the positive rate, it is necessary to increase the number of needles, which aggravates the pain of patients. Kevar et al. [16] called the condition as “cumulative pain”. Besides, the irregular growth of prostate cancer [17] increases the difficulty of locating the angle prostate-bladder-seminal vesicle. In addition, Nguyen et al. [18] found that apical biopsies were more painful than were biopsies from other areas of the prostate under PNB.

Hence, PNB cannot completely eliminate discomfort. A small number of nerve fibers are located on the anterior and superolateral parts of the prostate, and PNB does not block these nerve fibers, which is the limitation of PNB. Recently, some studies have shown that direct blocking of the origin of the prostatic nerves, the pelvic plexus, may obtained better analgesia [19–22].

In this study, we compared pelvic plexus block (PPB) with conventional PNB to evaluate whether PPB has better anesthetic efficacy in the process of TTPB.

## Methods

### Clinical data

The prospective, randomized controlled study was done in our hospital from July 2016 to August 2017. Ethics clearance was obtained from the Clinical Medical College of the Yangzhou University.

Study inclusion criteria included: digital rectal examination findings nodules; and/or ultrasound or magnetic resonance imaging revealings suspicious of prostate cancer; and/or PSA4 ~ 10 ng/ml with an abnormal free/total PSA or PSA density; and/or PSA > 10 ng/ml. Exclusion criteria were chronic pelvic pain syndrome, chronic prostatitis, previous prostate biopsies, allergy to local anesthetic, bleeding disorder, pathological rectum, active urinary tract infection and taking analgesic medications. All patients signed a written informed consent before prostate biopsy. Assisted by a computer program, an independent researchers used a random number table to randomly divide the patients into three groups. The patients of group-1 received prostate capsule local anesthesia (PLA), group-2 received PNB in addition to PLA, and group-3 received PLA along with a PPB. Regarding the grouping assignment, the patients were unaware the details.

### Biopsy procedure

The biopsy device included: A biplanar TRUS probe (Flex focus 1202; BK, Naerum, Denmark), brachytherapy stepping unit (Fixer, template, stepper) (Mick Radio-Nuclear Instruments, Mount Vernon, NY, USA), Bard biopsy gun (Bard MCl 820) and an 18-gauge biopsy needle.

Before prostate biopsy, complete blood cell count with differentiation, coagulation test and comprehensive metabolic panel were routinely performed. The patients were given oral intestinal clearance drugs one day before operation and emptied their stools on the morning of operation.

The patients were placed in a lithotomy position and underwent a conventional digital rectal examination. The TRUS probe was fixed on the brachytherapy stepping unit and was placed into the rectum. Then, the patients received the following anesthesia:

Group-1 (PLA): under the guidance of TRUS, the projection range of prostate in perineal skin was observed. In an area larger 0.5 cm than this range, infiltration anesthesia of perineal skin used 10 ml of 1% lidocaine. Then infiltration anesthesia was performed on the apex of the prostate. At the 1, 3, 5, 7, 9 and 11 o’clock of the area which prostate apex projected in the perineal skin, the syringe enters from these points and injected 12 ml of 1% lidocaine near the apex of the prostate.

**Fig. 1:**
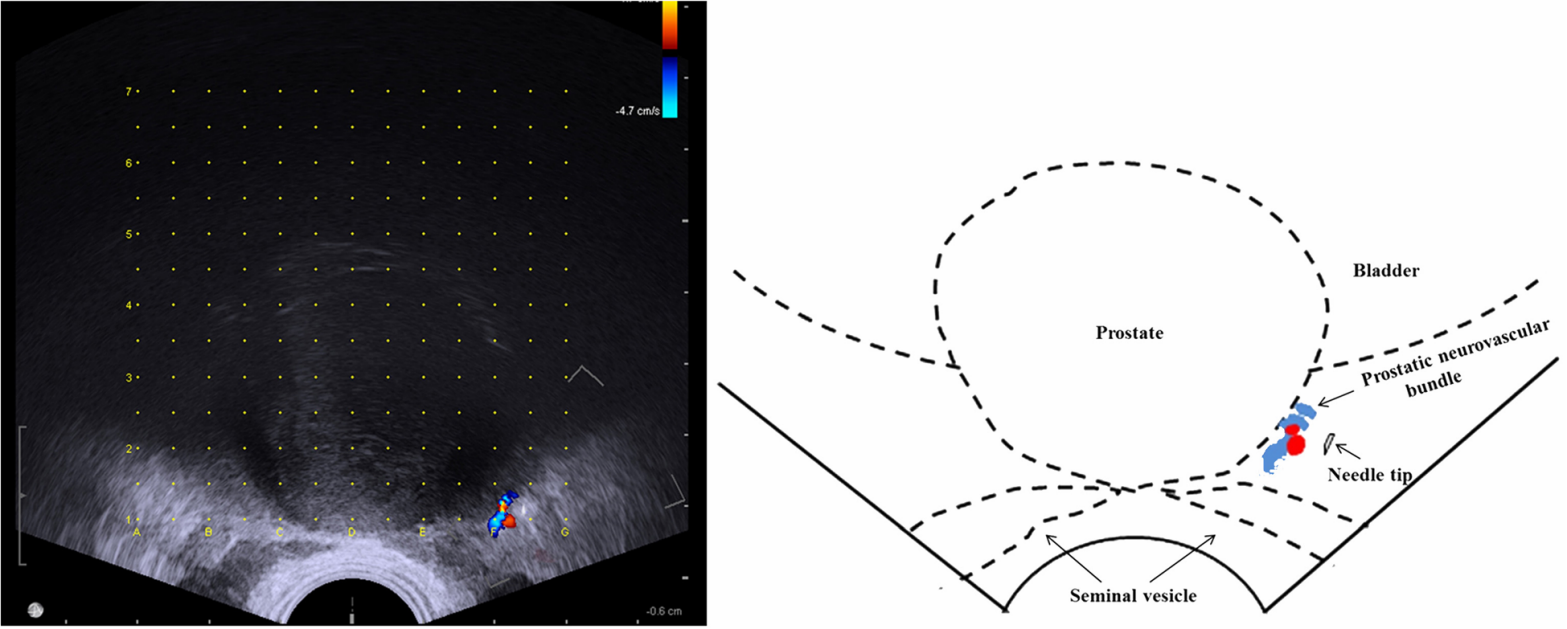
PNB: 5 ml of 1% lidocaine was injected into the position adjacent to the prostatic neurovascular bundle, location at the prostate-bladder-seminal vesicle angle

Group-2 (PLA + PNB): for PNB, by rotating the TRUS probe, the vessels in the prostatic neurovascular bundle (NVB) can be observed at the prostate-bladder-seminal vesicle angle. Therefore, the NVB can be located by blood vessels. In the region of NVB, ANSll 0.5 mm ∗ 112 mm spinal needle was used to inject 5 ml of 1% lidocaine to perform PNB. The same was done for the other side (Fig. 1).

**Fig. 2:**
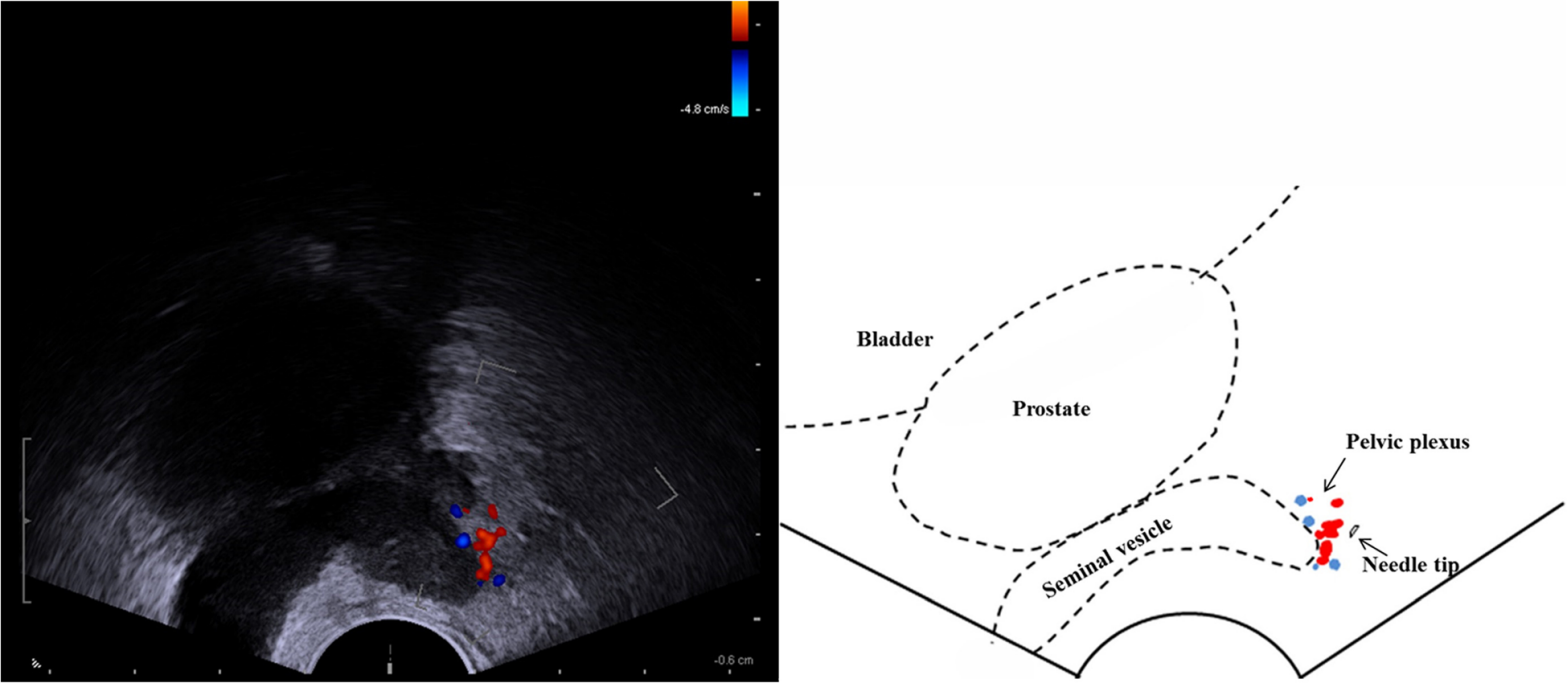
PPB: 5 ml of 1% lidocaine was injected into the region of the pelvic plexus, situated lateral to the apex of the seminal vesicles

Group-3 (PLA + PPB): for PPB, under color Doppler ultrasound guidance, pelvic plexus was positioned which situated in the lateral to the apex of bilateral seminal vesicles. Then, in the area of the pelvic plexus, 5 ml 1% lidocaine was injected (Fig. 2).

To avoid injecting local anesthetic into the vessels, the syringe was aspirated before injecting local anesthetic in the three groups. After 5 min, prostate biopsies were performed.

### Pain and complication assessment

Pain was evaluated by the visual analog scale (VAS; 0, none; 10, intolerable pain), and satisfaction was quantified using a visual numeric scale (VNS; 0, terrible; 4, perfect); only integers were allowed [23, 24]. Another independent resident and the patients were ‘blinded’ to the type of anesthesia given. The resident explained the VAS and VNS to the patients and requested them to rate their level of pain or satisfaction. VAS scores and VNS scores had two time points: VAS-1 and VNS-1 during the biopsy procedure, and VAS-2 and VNS-2 at 30 min after the procedure. The patients recorded the VAS and VNS scores without any other assistance.

Patients were given a nonvalidated self-administered questionnaire about complications such as hematuria, hemospermia, urinary retention and infection.

### Statistical analysis

Epi Info™ 7 (WHO, Geneva, Switzerland) was used for sample size calculation. 95% CI, 5% α error, 80% power and 90% population proportion were set. It was estimated that at least 42 men would be needed for each of the three groups. And according to the 20% predicted clinical missed follow-up rate, at least 51 patients in each group were required.

Data analysis was performed using SPSS (version 19.0; IBM Corp, Armonk, NY) statistical software. The VAS scores and VNS scores were shown as mean ± SD and were compared using the one-way ANOVA test. And the rates of complications were compared using *χ*^2^ test. *P* < 0.05 was considered statistically significant.

## Results

**Fig. 3:**
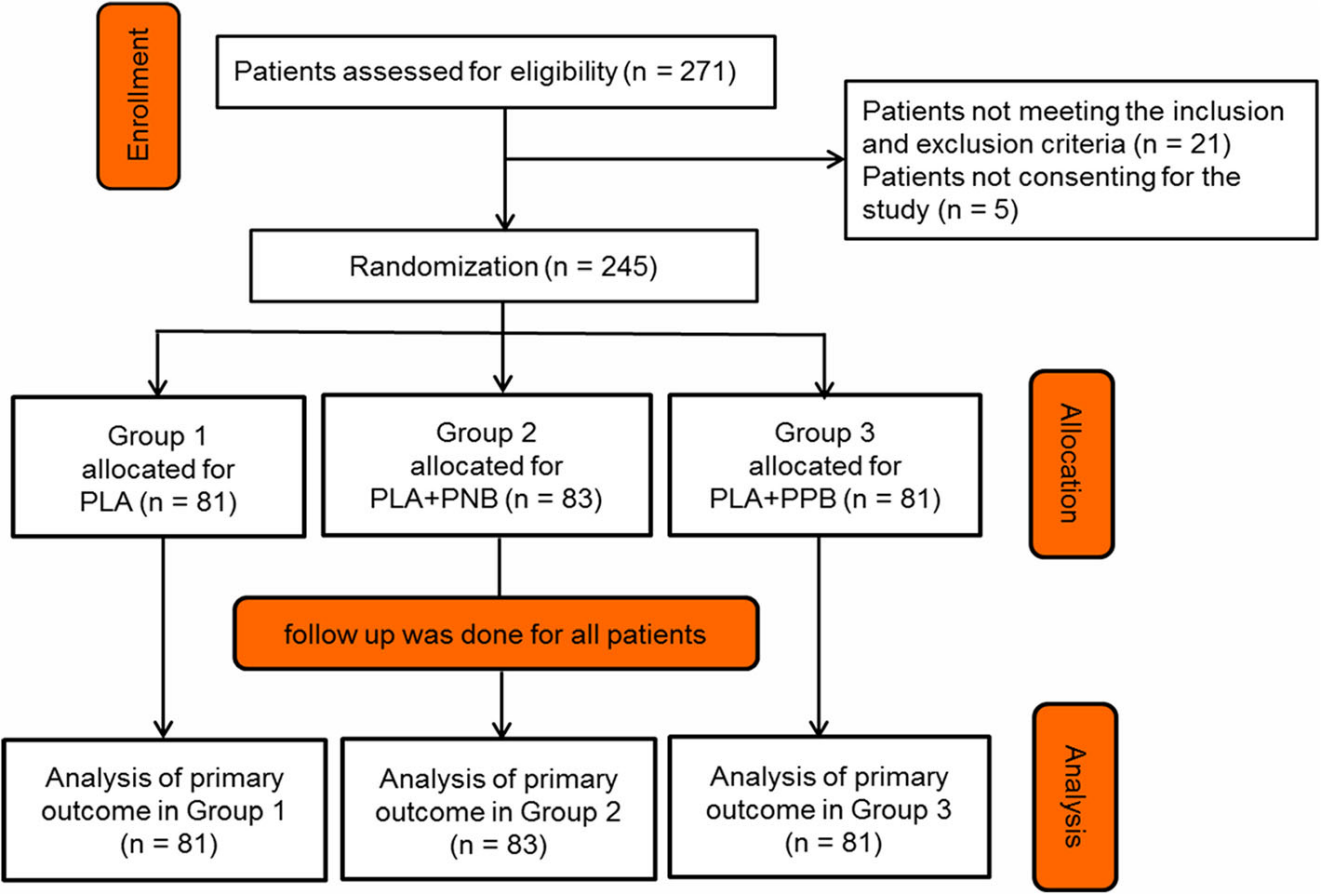
The flow diagram of the study as per the Consolidated Standard of Reporting Trials guidelines

From July 2016 to August 2017, 271 patients were performed TTPB in Clinical Medical College of Yangzhou University. Of these, 21 patients did not fulfil inclusion criteria and 5 did not provide consent. Ultimately, 245 patients were randomized into three groups. Groups-1, 2 and 3 included 81, 83 and 81 patients, respectively (Fig. 3). The age, prostate volume, serum PSA level and the number of cores showed no significant differences among these three groups, as presented in Table 1.

**Table 1:** The patients’ characteristics in the three groups

Groups	*N*	Age(years)	Prostate volume (ml)	PSA level (ng/ml)	Number of cores
Group-1	81	67.2 ± 9.5	47.7 ± 19.3	33.1 ± 23.1	23.3 ± 8.5
Group-2	83	68.2 ± 8.6	46.7 ± 20.4	30.9 ± 21.7	22.9 ± 8.2
Group-3	81	67.9 ± 9.1	48.2 ± 19.2	35.3 ± 22.6	23.1 ± 8.2
*P* value		0.790	0.885	0.475	0.945

In groups-1, 2 and 3, VAS-1 was 3.3 ± 1.6, 2.7 ± 1.3 and 2.1 ± 1.1, and VNS-1 was 2.8 ± 0.9, 3.1 ± 0.8 and 3.3 ± 0.2, respectively. The pain scores in group-3 were significantly lower than in group-2 (*P* = 0.003). Both the group-2 and group-3 outperformed the group-1 in alleviating pain. Satisfaction scores in group-3 were significantly higher than in group-2 (*P* = 0.039). Group-2 and group-3 were higher than group-1 in terms of satisfaction. In groups-1, 2 and 3, VAS-2 was 0.7 ± 0.4, 0.6 ± 0.5 and 0.5 ± 0.3, and VNS-2 was 3.5 ± 0.6, 3.5 ± 0.6 and 3.7 ± 0.8, respectively. At 30 min after the procedure, there were no statistically significant differences in VAS scores or VNS scores among the three groups (Table 2).

**Table 2:** The VAS scores and VNS scores in the three groups

Groups	Group-1 *n* = 81	Group-2 *n* = 83	Group-3 *n* = 81	*P* value		
				Group-1 vs 2	Group-1 vs 3	Group-2 vs 3
VAS-1	3.3 ± 1.6	2.7 ± 1.3	2.1 ± 1.1	0.007	0.000	0.003
VAS-2	0.7 ± 0.4	0.6 ± 0.5	0.5 ± 0.3	0.169	0.071	0.572
VNS-1	2.8 ± 0.9	3.1 ± 0.8	3.3 ± 0.2	0.027	0.000	0.039
VNS-2	3.5 ± 0.6	3.6 ± 0.3	3.7 ± 0.8	0.511	0.071	0.323

There was no significant difference in complications including hematospermia, hematuria, infection and urinary retention among the three groups (Table 3).

**Table 3:** The complications of prostate biopsy [% (cases)]

Groups	*N*	hematuria	urinary retention	infection	hemospermia
Group-1	81	32.1 (26)	3.7 (3)	2.5 (2)	3.7 (3)
Group-2	83	33.7 (28)	2.4 (2)	2.4 (2)	3.6 (3)
Group-3	81	30.9 (25)	2.5 (2)	1.2 (1)	4.9 (4)
*P* value		0.925	0.860	0.579	0.895

## Discussion

TTPB has become one of the most commonly methods for the diagnosis of Pca. As an invasive operation, TTPB is related to obvious discomfort and pain.

Currently, PNB is safe, easily performed and high-efficiency, and it is generally regarded as the preferred method for analgesia during biopsy needles inserting into the prostatic tissue [7–10]. Recently, PPB was reported to have a definitive theoretical advantage for decreasing pain, challenging conventional PNB [19–22].

The nerves of the prostate come from the pelvic plexus, including the sympathetic and parasympathetic nerves. The sympathetic nerve fibers are mainly transmitted to the pelvic plexus through the superior hypogastric plexus. Parasympathetic fibers originate from the 2–4 sacral spinal nerves and are transmitted through them. These nerves unite to form the pelvic plexus [21, 25]. And which is rectangular and 4 to 5 cm in length. The midpoint of pelvic plexus is located on the lateral to the apex of bilateral seminal vesicles. The pelvic plexus is penetrated by many vessels that supply the the prostate, seminal vesicles, bladder and rectum. At the most caudal portion of the pelvic plexus, the nerve innervating the prostate and the cavernosal nerves, the prostatic plexus, is generated [26, 27]. Subsequently, the prostatic plexus and the vessels of the prostate form the NVB, which is distributed along the posterolateral margin of the prostate and is considered to be the primary nerves innervating the prostate. However, there are a few other nerve fibers also running on the anterior and superolateral surfaces of the prostate [11, 19, 27, 28].

PNB mainly blocks the prostatic plexus located on the posterolateral side of the prostate, which is the primary nerve that supplies the prostate [19–22]. However, a small number of nerve fibers located on the anterior and superolateral parts of the prostate were omitted and thus not blocked. This may be the reason why a research has found that biopsies at the apex of the prostate were more painful than those at other sites [18]. PPB directly injects lidocaine into the pelvic plexus, and all nerve fibers supplying to the prostate are blocked. Hence, compared with PNB, PPB have theoretical advantages.

Wu et al. [22] first reported PPB. Under grayscale ultrasound guidance, they injected 5 ml of 1% lidocaine into lateral to the seminal vesicles apex. However, they didn’t find any reduce the pain associated with biopsy. Conversely, Akpinar et al. [21] found that compared with PNB, the analgesic efficacy of PPB (2 ml 2% lidocaine for each pelvic plexus) was better. Cantiello et al. [19] mixed 1% lidocaine with 0.75% Naropin and injected 2.5 ml mixture into each pelvic plexus lateral to the apex of the seminal vesicles under the guidance of color Doppler ultrasound. Jindal et al. [20] injected 2.5 ml 2% lidocaine into the bilateral pelvic plexus. In studies of Cantiello et al. and Jindal et al., they reported PPB had a better anesthetic effect than did PNB. The results of the last three researches are different from those of Wu et al., and the reason may be related to the use of color Doppler ultrasound, which can provide more accurate positioning of pelvic plexus.

All of the above studies were meant to explore the anesthetic efficacy of PPB in the transrectal prostate biopsy. Owing to the high positive rate and few complications, The value of TTPB in the diagnosis of Pca is becoming more and more important [2, 3]. Hence, we evaluated the anesthetic efficacy of PPB in TTPB in this study. Our present outcomes showed that the PPB group had higher satisfaction and lower pain scores than did the PNB group. Both these groups had higher levels of satisfaction, and pain control was better than that in the group without any nerve block. After 30 min of the biopsy, most patients returned to normal without pain. There were no differences in pain scores or satisfaction scores at 30 min after the procedure among the three groups.

Doppler ultrasound can avoid damage to the vessels and accurately inject the local anesthetics into the region of the pelvic plexus, thus reducing the incidence of complications related to the nerve block anesthesia technique, especially vascular complications. In our study, there was no significant difference in complications including hematospermia, hematuria, infection and urinary retention among the three groups .

Our study has several limitations. We assessed pain scores and satisfaction scores after biopsy. This might lead to ‘recall bias’. Nevertheless, we chose this method for avoiding any possible factors for disturbing biopsy performed by the resident, which may affect the scores. Although VAS and VNS are relatively objective indicators of pain detection, the evaluation of pain is still dominated by the subjective feelings of patients, and there is a lack of objective quantitative indicators, which needs further study. And in the future, we would study whether PPB results in more adhesions, making the surgical steps more difficult. We also need to explore whether it affects erectile function and urinary continence.

## Conclusions

Under the guidance of color Doppler ultrasound, PPB has better analgesic efficacy than did PNB in TTPB process and both were superior to no nerve block.

## Data Availability

The datasets used and/or analysed during the current study are available from the corresponding author on reasonable request.

## Abbreviations

Pca: Prostate cancer
PLA: Prostate-coated local anesthesia
PPB: Pelvic plexus block
PSA: Prostate specific antigen
PSAD: Prostate specific antigen density
TRUS: Transrectal ultrasound
TTPB: Transperineal template-guided prostate biopsy
VAS: Visual analogue scale
VNS: Visual numeric scale

## References

[1] Heijnsdijk EA, Wever EM, Auvinen A, Hugosson J, Ciatto S, Nelen V, Kwiatkowski M, Villers A, Paez A, Moss SM (2012). Quality-of-life effects of prostate-specific antigen screening. N Engl J Med.

[2] Muthuveloe D, Telford R, Viney R, Patel P (2016). The detection and upgrade rates of prostate adenocarcinoma following transperineal template-guided prostate biopsy - a tertiary referral Centre experience. Central European journal of urology.

[3] Mai Z, Xiao Y, Yan W, Zhou Y, Zhou Z, Liang Z, Ji Z, Li H. Comparison of lesions detected and undetected by template-guided transperineal saturation prostate biopsy. BJU Int. 2017. 10.1111/bju.13977 28771912

[4] Udeh EI, Amu OC, Nnabugwu II, Ozoemena O (2015). Transperineal versus transrectal prostate biopsy: our findings in a tertiary health institution. Niger J Clin Pract.

[5] Bingqian L, Peihuan L, Yudong W, Jinxing W, Zhiyong W (2009). Intraprostatic local anesthesia with periprostatic nerve block for transrectal ultrasound guided prostate biopsy. J Urol.

[6] Ukimura O, Coleman JA, de la Taille A, Emberton M, Epstein JI, Freedland SJ, Giannarini G, Kibel AS, Montironi R, Ploussard G (2013). Contemporary role of systematic prostate biopsies: indications, techniques, and implications for patient care. Eur Urol.

[7] Anup K, Pawan V, Niraj K, Biswajit N, Nayan MK (2013). A prospective randomized trial comparing three different analgesic techniques for pain control during transrectal ultrasound guided prostate biopsy: a single center experience. Minerva urologica e nefrologica = The Italian journal of urology and nephrology.

[8] Otunctemur A, Dursun M, Besiroglu H, Can Polat E, Cakir SS, Ozbek E, Karadeniz T (2013). The effectivity of periprostatic nerve blockade for the pain control during transrectal ultrasound guided prostate biopsy. Archivio italiano di urologia, andrologia : organo ufficiale [di] Societa italiana di ecografia urologica e nefrologica / Associazione ricerche in urologia.

[9] Wang J, Wang L, Du Y, He D, Chen X, Li L, Nan X, Fan J (2015). Addition of intrarectal local analgesia to periprostatic nerve block improves pain control for transrectal ultrasonography-guided prostate biopsy: a systematic review and meta-analysis. Int J Urol.

[10] Wang N, Fu Y, Ma H, Wang J, Gao Y (2016). Advantages of caudal block over intrarectal local anesthesia plus periprostatic nerve block for transrectal ultrasound guided prostate biopsy. Pakistan journal of medical sciences.

[11] Nash PA, Bruce JE, Indudhara R, Shinohara K (1996). Transrectal ultrasound guided prostatic nerve blockade eases systematic needle biopsy of the prostate. J Urol.

[12] Conde Redondo C, Alonso Fernandez D, Robles Samaniego A, Del Valle Gonzalez N, Castroviejo Royo F, Delgado Marcos C, Rodriguez Toves A, Martinez-Sagarra Oceja JM (2006). TRUS-guided biopsy: comparison of two anesthetic methods. Actas Urol Esp.

[13] Hergan L, Kashefi C, Parsons JK (2007). Local anesthetic reduces pain associated with transrectal ultrasound-guided prostate biopsy: a meta-analysis. Urology.

[14] Luan Y, Huang TB, Gu X, Zhou GC, Lu SM, Tao HZ, Liu BD, Ding XF (2016). Effect of prostate volume on the peripheral nerve block anesthesia in the prostate biopsy: a strobe-compliant study. Medicine.

[15] Leibovici D, Shilo Y, Raz O, Stav K, Sandbank J, Segal M, Zisman A (2013). Is the diagnostic yield of prostate needle biopsies affected by prostate volume?. Urol Oncol.

[16] Kaver I, Mabjeesh NJ, Matzkin H (2002). Randomized prospective study of periprostatic local anesthesia during transrectal ultrasound-guided prostate biopsy. Urology.

[17] Konyalioglu E, Tarhan H, Cakmak O, Pala EE, Zorlu F (2015). Prostate cancer volume estimations based on transrectal ultrasonography-guided biopsy in order to predict clinically significant prostate cancer. Int Braz J Urol.

[18] Nguyen CT, Jones JS (2007). Comparison of traditional basal and apical periprostatic block: impact on injection pain and biopsy pain. BJU Int.

[19] Cantiello F, Cicione A, Autorino R, Cosentino C, Amato F, Damiano R (2012). Pelvic plexus block is more effective than periprostatic nerve block for pain control during office transrectal ultrasound guided prostate biopsy: a single center, prospective, randomized, double arm study. J Urol.

[20] Jindal T, Mukherjee S, Sinha RK, Kamal MR, Ghosh N, Saha B, Mitra N, Sharma PK, Mandal SN, Karmakar D (2015). Transrectal ultrasonography (TRUS)-guided pelvic plexus block to reduce pain during prostate biopsy: a randomised controlled trial. BJU Int.

[21] Akpinar H, Tufek I, Atug F, Esen EH, Kural AR (2009). Doppler ultrasonography-guided pelvic plexus block before systematic needle biopsy of the prostate: a prospective randomized study. Urology.

[22] Wu CL, Carter HB, Naqibuddin M, Fleisher LA (2001). Effect of local anesthetics on patient recovery after transrectal biopsy. Urology.

[23] Robins D, Lipsky M, RoyChoudry A, Wenske S (2018). Assessment of discomfort and pain in patients undergoing fusion magnetic resonance imaging-guided vs TRUS-guided prostate biopsy. Urology.

[24] Doganca T, Savsin A, Erdogan S, Altindas F, Ozdemir F, Ekici B, Obek C (2015). Procedural sedation and analgesia as an adjunct to periprostatic nerve block for prostate biopsy: a prospective randomized trial. J Clin Ultrasound.

[25] Choi JW, Kim WH, Lee CJ, Sim WS, Park S, Chae HB. The optimal approach for a superior hypogastric plexus block. Pain practice : the official journal of World Institute of Pain. 2017. 10.1111/papr.12603 28520297

[26] Schlegel PN, Walsh PC (1987). Neuroanatomical approach to radical cystoprostatectomy with preservation of sexual function. J Urol.

[27] Walsh PC, Lepor H, Eggleston JC (1983). Radical prostatectomy with preservation of sexual function: anatomical and pathological considerations. Prostate.

[28] Hollabaugh RS, Dmochowski RR, Steiner MS (1997). Neuroanatomy of the male rhabdosphincter. Urology.

